# Robustness of speech intelligibility at moderate levels of spectral degradation

**DOI:** 10.1371/journal.pone.0180734

**Published:** 2017-07-05

**Authors:** Sierra Broussard, Gregory Hickok, Kourosh Saberi

**Affiliations:** Department of Cognitive Sciences, University of California, Irvine, Irvine, California, United States of America; University of Kent, UNITED KINGDOM

## Abstract

The current study investigated how amplitude and phase information differentially contribute to speech intelligibility. Listeners performed a word-identification task after hearing spectrally degraded sentences. Each stimulus was degraded by first dividing it into segments, then the amplitude and phase components of each segment were decorrelated independently to various degrees relative to those of the original segment. Segments were then concatenated into their original sequence to present to the listener. We used three segment lengths: 30 ms (phoneme length), 250 ms (syllable length), and full sentence (non-segmented). We found that for intermediate spectral correlation values, segment length is generally inconsequential to intelligibility. Overall, intelligibility was more adversely affected by phase-spectrum decorrelation than by amplitude-spectrum decorrelation. If the phase information was left intact, decorrelating the amplitude spectrum to intermediate values had no effect on intelligibility. If the amplitude information was left intact, decorrelating the phase spectrum to intermediate values significantly degraded intelligibility. Some exceptions to this rule are described. These results delineate the range of amplitude- and phase-spectrum correlations necessary for speech processing and its dependency on the temporal window of analysis (phoneme or syllable length). Results further point to the robustness of speech information in environments that acoustically degrade cues to intelligibility (e.g., reverberant or noisy environments).

## Introduction

Phase spectrum analysis is often ignored in models of auditory spectral processing in humans despite our knowledge that humans are not phase deaf when listening to complex sounds. Phonemes, for example, are most often represented as a structural component of the amplitude spectrum [1–2]. However, a number of studies have found that phase plays a major role in speech analysis and recognition. Oppenheim and Lim [3] found evidence through informal experiments that phase information could be useful in speech-signal reconstruction for long signal times, concluding that changing the phase spectrum of a speech sound can alter its phonetic value.

Humans are able to identify vowels using only phase spectrum information at low fundamental frequencies, and speech comprehension has been shown to be more dependent on long-term phase spectrum than amplitude-spectrum information [2, 4, 5]. Liu and colleagues [2], for example, investigated the impact of the phase spectrum on stop consonants and found that it is used to determine voicing properties and is critical for setting the structure of formant transitions. Phase information is also more important for consonants with strong burst releases than weak burst releases. Another study found similar results using full sentence stimuli [1]. Phase degradation has also been reported to make speech in noise recognition more difficult [6], however the interpretation of this finding is confounded by the methods employed, as adding noise to speech whose phase spectrum has been degraded by a preset value, will further degrade the phase spectrum, resulting in inaccurate measures of the effects of phase-spectrum degradation on intelligibility.

A critical question is the effect of the temporal window of spectral analysis on the relative contribution of amplitude and phase spectra to speech intelligibility. Several studies have shown that the type of spectral information that best maintains intelligibility varies by window length [1, 2]. It has been shown that for phoneme length (<128 ms) time windows, amplitude information is most useful to intelligibility. However, at longer (>128 ms) window lengths, phase-spectrum information is more important. This 128 ms crossover point falls almost exactly between the average durations of phonemes and syllables, which have been suggested as basic segments of analysis in speech processing [7]. The average lengths of these speech units are ~30 ms and ~250 ms, respectively, and recent EEG and MEG research has presented evidence of a neural basis for these two window sizes in speech perception [8–12]. These studies have shown that the auditory cortex prefers stimuli with temporal modulations at gamma-band (~20–80 ms) and theta-band (~150–300 ms) rates, suggesting that these may represent some form of neural parsing or temporal integration [8].

Temporal envelope, fine structure, and periodicity each contribute different types of cues to speech intelligibility [13]. Phonemes are identified by a combination of voicing, manner, and place of articulation. Information about voicing and manner of articulation appear in all three of the previously mentioned signal components. Manner and voicing cues appear in envelope information as differences in rise times (as in ‘*ch*ip’ and ‘*sh*ip’), long periods of high amplitude for vowels, or as brief silent gaps to indicate a voiceless plosive [14–16]. Aperiodicity and high-frequency fine-structure cues can signal that a sound is either voiceless or a fricative [17]. Place of articulation is determined by the frequency spectrum of initial release bursts and consecutive formants, which is information found in fine structure [18, 19]. Tempo and stress help to parse sentences and distinguish between certain types of words (such as *re*bel and re*bel*). These parsing cues are only found in periodicity and temporal envelope information. While gaps of silence in the temporal envelope do not necessarily demarcate word boundaries, tempo is still a helpful envelope cue for segmenting words. Similarly, tempo can provide weak cues for vowel identity due to the covariance of vowel length and vowel quality [20]. Periodicity is the prime correlate of vocal pitch because it represents the rate of vocal fold vibration. Patterns of vocal pitch provide the primary cues used to indicate which words and syllables are stressed; these are extremely important cues to word identity in tonal languages such as Chinese. However, increases in the amplitude of temporal envelope also play a small role in marking stress [21].

Most recent studies on speech intelligibility have focused on the temporal envelope modulations of speech signals. Several studies have demonstrated that, as long as the signal’s narrowband temporal envelopes are adequately preserved, a speech signal will be intelligible regardless of how the speech spectrum information is altered [22–24]. It is argued that speech is made less intelligible by degrading information in one or both spectral domains (amplitude or phase), mainly because the temporal envelope is also degraded by these manipulations. By modelling the outputs of peripheral filters, one group of researchers determined that the intelligibility of spectrally degraded stimuli was highly correlated with narrowband envelope preservation [1]. These findings suggest that the necessary spectral information for intelligibility is ultimately dependent on the type of information that best preserves the temporal envelope.

Naturalistic speech environments, however, are best represented by intermediate spectral correlation values since amplitude and phase spectra of a signal will both be partially degraded in a noisy or reverberant environment. All prior findings in this area of research are based on stimuli with only one type of spectral component preserved, usually achieved by separately decorrelating to zero either the amplitude or phase spectrum relative to the original waveform. Thus, the resulting stimuli maintain either the original amplitude or phase spectrum only, while the other spectral component is usually replaced with noise [1, 2, 25, 26].

The purpose of this study is to investigate the relative contributions of phase and amplitude spectra on sentence intelligibility by independently decorrelating, to various degrees, their amplitude and phase spectra relative to those of the original sentence across several time-window sizes. Investigating intelligibility using intermediate phase and amplitude correlation values (between 0 and 1) will allow a better understanding of their individual and joint influence on speech perception. Furthermore, these results will provide intelligibility scores for a larger variety of degraded *temporal envelopes*, allowing an in-depth analysis of the relationship between spectral and temporal representations of speech stimulus.

## Methods

### Participants

Informed written consent was obtained from all participants. Fifteen adult listeners participated in the study (6 females, Mean age = 25 years, σ = 2.2). All participants had normal hearing and were native English speakers. None were familiar with the sentences in the Hearing in Noise Test (HINT) database [27]. Subjects were recruited through IRB-approved postings on campus and through word of mouth starting in 2013 and continuing through 2016. Some had participated in prior experiments and had indicated an interest to participate in the current study. No subjects dropped out of the study or were excluded from data analysis. This study was approved by the IRB of the University of California, Irvine (HS# 2010–7679).

### Stimuli

**Fig 1:**
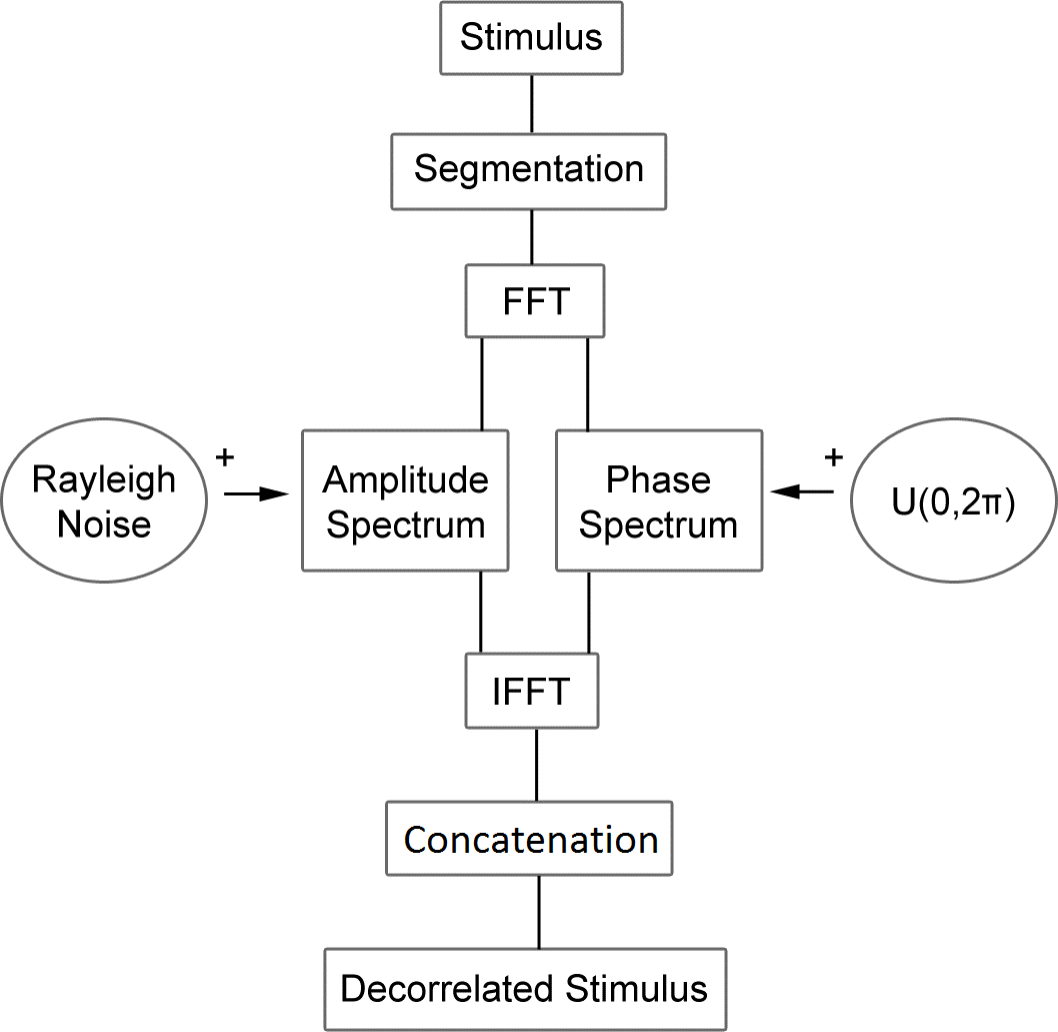
Decorrelation method. Diagram of the method used to decorrelate speech stimuli. Each sentence was divided into segments of equal duration. Each segment was then Fourier transformed, yielding separate amplitude and phase spectra. The phase and amplitude spectra were then independently decorrelated relative to the original by a specific amount. Segments were then inverse Fourier transformed, and concatenated in their original temporal order to form a degraded sentence.

Each stimulus was created by taking a sentence from the HINT database and adding noise through a decorrelation process (Fig 1). First, the sentence was divided into one of three time-window sizes: 30 ms, 250 ms, or equal to the duration of the sentence. Each segment was then Fourier transformed, yielding separate amplitude and phase spectra. These spectra were then separately decorrelated relative to the original by a specific amount. The decorrelation process had several stages. First, for amplitude-spectrum decorrelation, we added to each amplitude component in the frequency domain, a random number selected from a Rayleigh distribution. A Rayleigh distribution was selected because the amplitude components of Gaussian noise in the frequency domain are Rayleigh distributed. The vector containing the amplitude-spectrum values of the speech sound was added, on a point-by-point (bin by bin) bases, to a vector of the same size containing the random numbers from the noise distribution (with appropriate adjustments for negative frequency components):

$$a^{\prime }(f)=k*n(f)+(1-k)*a(f)$$ (1)

where a(f) is the amplitude-spectrum vector as a function of frequency, n(f) is the noise vector, a’(f) is the new, decorrelated amplitude spectrum, and k is a scalar. We then measured the Pearson product-moment correlation value (*r*_*α*_) between a(f) and a’(f). When k = 0, the correlation between the new and original amplitude spectrum of speech (*r*_*α*_) is 1 (full correlation). When k = 1, the amplitude spectrum of speech is fully replaced with that of Gaussian noise, and the correlation is zero. For in-between values (moderate correlation values), we first generated a k-to-r transfer function that provided an initial estimate of how the values of k are associated with specific correlation values (between original and degraded amplitude spectrum of speech). This was done by incrementally adding noise (i.e., increasing value of k) to the amplitude-spectrum of several speech sentence and measuring the resulting correlation. The transfer function was saved and served as an initial starting point for determining the relation between k and r on each trial. On any given trial of the experiment, a speech segment was decorrelated by adding noise to the amplitude spectrum as described above, and fine tuning the value of k iteratively in a loop till the desired correlation between a’(f) and a(f) was achieved within a tolerance limit of smaller than 0.01. This was done for every segment of each speech sentence upon the presentation of that sentence. A similar procedure was used for decorrelating the phase spectrum with the following differences: 1) phase noise was selected from a 0–2π uniform distribution; 2) the correlation measured was not the linear Pearson value, but a circular statistical correlation value that has the same properties as a linear Pearson, but takes into account the circular nature of phase wrapping [28, 29]. We will refer to this phase correlation value as *r*_θ_. Each segment was then inverse Fourier transformed to the time domain, it’s RMS level matched to the original segment’s RMS, its start and end points smoothed with a ~4 ms linear rise-decay ramp (100 samples at 22.05 kHz) to reduce spectral splatter at transition points between segments in a sentence, and then concatenated with other segments in their original order to generate the degraded sentence. The entire decorrelation process took less than 1 second and was done between trials of a run.

We paired each of the 3 amplitude-spectrum correlation values (0, 0.5, 1) with each phase-spectrum correlation value (0.4, 0.6. 0.8, 1), creating 12 unique (amplitude x phase) conditions. Based on pilot data we determined that these values would be most informative for investigating intelligibility as they provided a wide range of performance levels. Because we were particularly interested in looking at the effects of the phase spectrum, as it has not been studied as extensively as the effects of the amplitude spectrum on intelligibility, we selected a greater number of phase spectrum values. Our pilot study showed that the lower bound of 0.4 for phase-spectrum correlation is adequate since participants were unable to identify any words when the phase-spectrum correlation was below this value. All stimuli were played through HD380 Pro Sennheiser headphones at a sampling rate of 22.05 kHz at an average level of approximately 70 dB SPL (A weighted) measured using a 6-cc coupler, 0.5-inch microphone, and a Precision Sound Analyzer (Brüel & Kjær, Model 2260).

### Procedure

Sentences from the HINT database were randomly assigned to each condition and presented to participants in a random order. No sentence was presented more than once per participant. Each subject participated in only one of the three temporal window condition (30 ms, 250 ms, or full length sentence), resulting in a 3 (amplitude correlation) x 4 (phase correlation) x 3 (time window size) mixed-measures experimental design. Five subjects were assigned to each of the three temporal-window conditions, and each subject participated in one experimental session which comprised two blocks of 60 trials that lasted approximately 30 minutes. This resulted in 10 sentences (~40 words) per condition.

The experiment was conducted in a double-walled anechoic chamber (Industrial Acoustics Company). Participants were seated at a computer and instructed to listen to each sentence and type as many words as they could understand, ignoring punctuation. Because sentences are semantically meaningful, it is possible that context may provide some cue to word identification. However, use of sentence material to study intelligibility under acoustically degraded conditions is standard practice as such sentences (instead of isolated words) are the type of stimuli most encountered in natural settings. The HINT corpus for example has been used in hundreds of speech intelligibility studies. In addition, subjects were instructed to report words that they were confident about even if it did not make sense semantically because a participant may have misheard an earlier word in the sentence.

There was no time limit for each trial, so participants’ typing speed did not affect their ability to perform the task. An experimental run began with 10 practice sentences which were repeated until the subject reported feeling comfortable with the interface and task. The sentences were scored based on individual correct keywords. Potentially confusing verbs (“are/were”), pronouns (“he/she”), prepositions (“in”), conjunctions (“or”), and articles (“the”) were excluded from scoring. Average sentence length including non-keywords was 5.3 words, which dropped to 4.1 after exclusions. Total number of correct keywords was compared to total number of keywords for each condition to determine the percent correct for each run. This number represented the degree of intelligibility.

## Results

**Fig 2:**
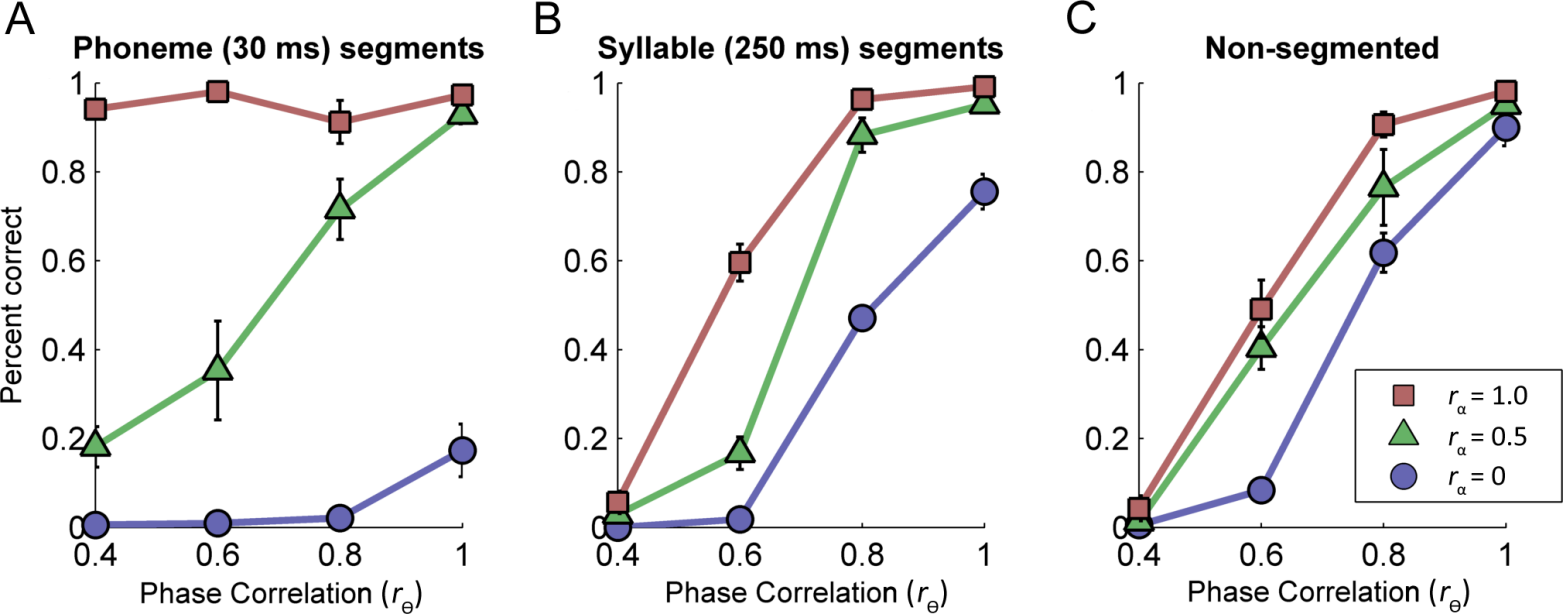
Contribution of phase and amplitude spectra to intelligibility. Speech intelligibility as a function of phase- and amplitude-spectrum correlation relative to those of the original unaltered sentence (r_θ_ and r_α_, respectively). Each panel depicts results for one of the three temporal window sizes. Each point is calculated from ~40 words per subject. Error bars represent +/- 1 standard error of the mean.

Fig 2 shows average intelligibility scores for each window size as a function of amplitude- and phase-spectrum correlations. Each point is based on 10 sentences (~40 words) per listener (~200 words per point). An intelligibility score of 1 indicates that every subject correctly identified all keywords in all sentences for that condition.

A 3 (amplitude correlation) x 4 (phase correlation) x 3 (time window size) mixed-measures ANOVA showed a significant main effect of amplitude-spectrum correlation (*F*(2,24) = 349.21, *p* < .01) and a significant main effect of phase-spectrum correlation (*F*(3,36) = 1231.61, *p* < .01). No main effect of window size was found (*F*(2,12) = .92, *p* = .42), but there were significant interaction effect between amplitude-spectrum correlation and window size (*F*(4,24) = 67.94, *p* < .01), as well as between phase-spectrum correlation and window size (*F*(6,36) = 110.69, *p* < .01). These results suggest that both the effect of amplitude and phase spectrum correlations on speech intelligibility varied by window size. Finally, there was a significant three-way interaction (*F*(12,72) = 9.28, *p* < .01), suggesting that the interaction between phase and amplitude correlations was different at different window sizes.

### Effects of decorrelation on non-segmented conditions

A 3 (amplitude correlation) x 3 (phase correlation) mixed-measures ANOVA was used to compare the effects of decorrelations on this window size. Note that one of the phase conditions (0.4) was removed from analysis because as shown in Fig 2A, intelligibility scores converged to zero at this correlation value even for an amplitude-spectrum correlation of 1. We therefore removed this point from the ANOVA to avoid a misleading significant interaction effect. Both a main effect of amplitude and phase correlation was found (*F*(2,8) = 59.11, *p* < .05; *F*(2,8) = 352.69, *p* < .01, respectively). A significant interaction was not observed (*F*(4,16) = X = 2.64, *p* = .07), suggesting that adding phase information did not improve intelligibility more for one level of amplitude correlation than another.

### Effects of decorrelation on the 250-ms (syllable length) conditions

A second 3 x 3 mixed-measures ANOVA was calculated to determine the effects of decorrelations on intelligibility specifically for the 250-ms time-window conditions. Similar to the full-length window, there were main effects of both amplitude and phase correlations (*F*(2,8) = 751.13, *p* < .05; *F*(2,8) = 574.87, *p* < .01, respectively). Unlike the full-length time-window condition, there was a significant interaction effect between amplitude and phase correlations (*F*(4,16) = 14.44, *p* < .01). As seen in Fig 2B, when amplitude information is partially corrupted (*r*_α_ = 0.5), increasing phase-spectrum correlation from 0.6 to 0.8 improves intelligibility scores considerably more than that at other amplitude-spectrum correlations (0 and 1).

### Effects of decorrelation on the 30-ms (phoneme length) conditions

Unlike in the previous two window sizes, there was no point of convergence for the 30 ms time-window conditions. Because of this, the 0.4 phase correlation value, which was excluded from analysis as a floor performance level in the prior two conditions (syllable and full length windows), was included in the statistical analysis of the phoneme-length conditions. A 3 x 4 mixed measures ANOVA showed a main effect of both amplitude- and phase-spectrum correlations (*F*(2,8) = 167.26, *p* < .01; *F*(8,3) = 61.12, *p* < .01, respectively). A significant interaction effect was also observed *F*(6,24) = 19.54, *p* < .01) but the form of this interaction is dissimilar to that seen for the 250 ms condition (compare panels B and C of Fig 2).

## Discussion

### Speech intelligibility for intermediate correlation values

At the most extreme correlation values (0 and 1) our results are consistent with previous studies that have investigated the effects of spectral decorrelation [1, 2, 25, 26]. However, real speech rarely occurs under perfect conditions, and it is implausible for only one type of spectral component to be degraded outside of laboratory conditions. Therefore, partially degraded amplitude and phase conditions may more accurately represent naturalistic speech environments.

In general, collapsing across window sizes, intelligibility was more adversely affected by phase-spectrum decorrelation than by amplitude-spectrum decorrelation even though both affected intelligibility to some degree. For longer window conditions, when the phase-spectrum was decorreled to 0.4, speech became unintelligible (Fig 2 panels A and B). The one phase-condition under which intelligibility seemed unaffected was for *r*_α_ = 1 at the shortest time window of 30ms (red square symbols of Fig 2C). Conversely, when phase-spectrum information is left intact (*r*_θ_ = 1) amplitude-spectrum decorrelation has little impact on intelligibility, except for one case, the shortest time window when *r*_α_ = 0 (blue circles in the Fig 2C). If the phase information is left intact, decorrelating the amplitude spectrum to intermediate values has no effect on intelligibility. If the amplitude information is left intact, decorrelating the phase spectrum to intermediate values significantly degrades intelligibility for the longer time windows.

Interestingly, at the short time window (30 ms), phase cues clearly have a major impact on performance at the intermediate amplitude-spectrum correlation (green line, Fig 2C). This novel finding is contrary to predictions of prior work that suggests little effect of the phase spectrum at short (phoneme length) time windows. Overall, intermediate correlation values show a significant monotonic effect of phase-spectrum correlation on intelligibility at all time windows (i.e., window size does not matter), a small monotonic effect of amplitude-spectrum correlation for the long time windows and a non-monotonic (interaction) effect of amplitude-spectrum correlation for the short time window.

### Equal intelligibility contours

**Fig 3:**
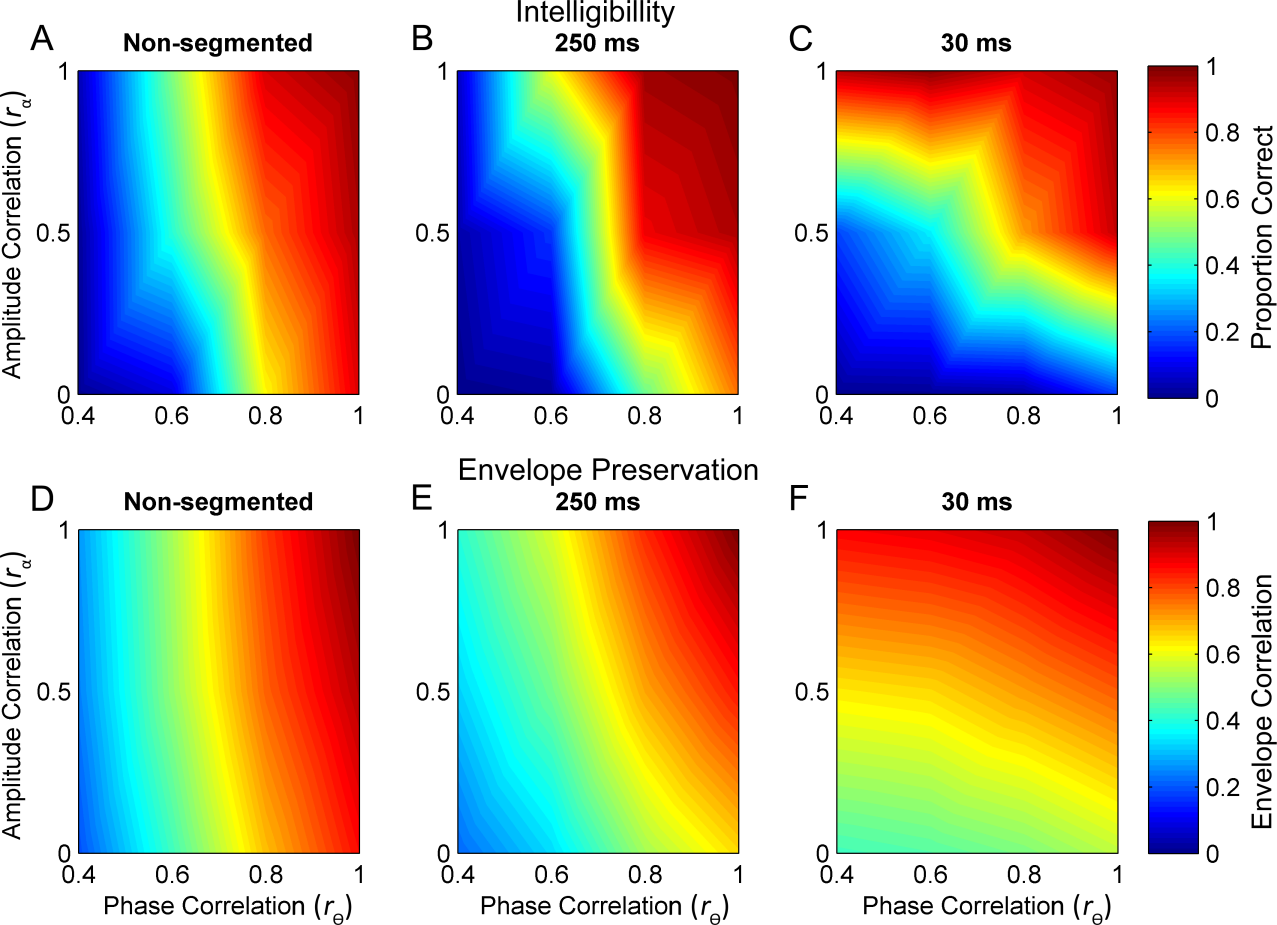
Comparison of equal intelligibility contours with envelope correlations. Top row shows equal intelligibility contours as a function of phase- and amplitude-spectrum correlation (r_θ_ and r_α_, respectively). A score of 1.0 (dark red) represents perfect intelligibility while dark blue represents an intelligibility score of zero. (A-C) Equal-correlation contours shown for each of the three window sizes. (D-F) These are the correlations between the temporal envelopes of two types of stimuli centered at 1 kHz: the original unaltered sentences and the same sentences whose phase and amplitude spectra were decorrelated by the values shown along the x-y axes. A score of 1.0 (dark red) represents perfect correlation between the altered and unaltered envelopes while 0 correlation is represented by dark blue.

As noted above, in general, the effect of amplitude-spectrum decorrelation increases as window size decreases. Conversely, the effects of phase-spectrum correlation increase as window size increases, but only for extreme correlation values (0 and 1). At an intermediate amplitude-spectrum correlation (*r*_α_ = 0.5), phase effects seem to be relatively independent of window size (green lines). Our findings suggest, that at least in some cases, there is a tradeoff between the importance of the two cues as a function of temporal window size, though this tradeoff is not necessarily linear. These findings further suggest that there are various combinations of *r*_θ_ and *r*_α_ that give rise to sets of equal intelligibility contours. Top row of Fig 3 shows these contours for the three time windows. A score of 1.0 (dark red) represents perfect intelligibility while dark blue represents an intelligibility score of zero. Note how the slopes of the equal-intelligibility contours increase with window size. The bottom panels of Fig 3 show equal-correlation contours between the temporal envelopes of two types of stimuli: 1) the original unaltered sentences and, 2) the same sentences whose phase and amplitude spectra were decorrelated by the values shown along the x-y axes.

**Fig 4:**
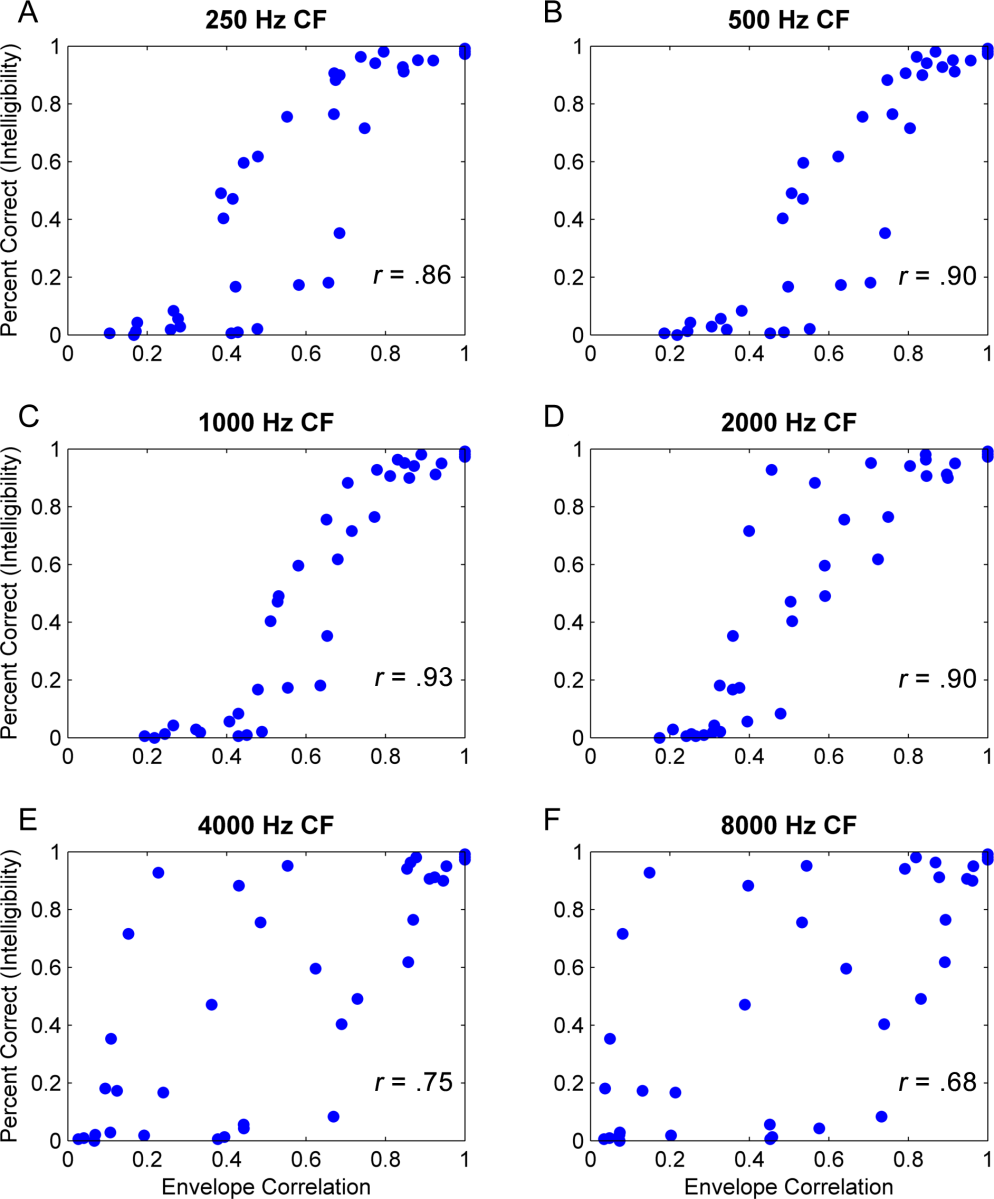
Narrow-band envelope correlations. Envelope correlations are calculated by comparing the narrow-band envelopes of normal (unaltered) stimuli and the corresponding decorrelated envelopes. Each point represents a single condition. Each frequency band determined by a 1/3 octave narrowband Gammatone filter. These correlations were calculated using the average values of all sentences in the HINT database. The correlation value between each frequency band envelope and intelligibility is depicted on the corresponding panel.

Note that the bottom panels do not show intelligibility scores (or any other behavioral measure). Rather they show the correlation between the narrowband envelopes of the unaltered and decorrelated sentences, at the output of a filter centered at 1 kHz (simulating the output of a cochlear filter). The reason for filtering at 1 kHz is that, first, the auditory system processes these waveforms not as broadband sounds, but through cochlear filters, and second, because our analysis below (Fig 4) demonstrates that the intelligibility performance is best predicted by examining information near the 1-kHz band.

These envelope correlations were calculated using the average values of all sentences in the HINT database. The similarity between equal intelligibility contours (top panels) and equal envelope-correlation contours (bottom panels) suggests that one major cue to intelligibility may be the narrowband temporal envelopes which are degraded more precipitously with phase-spectrum decorrelation than with amplitude-spectrum decorrelation.

Fig 4 makes this point clearer by plotting intelligibility scores, collapsed across window sizes, as a function of temporal envelope correlations (i.e., the correlation between the temporal envelopes of the altered and unaltered waveforms at the output of narrowband filters). Each panel shows this analysis for a different filter center frequency: 250, 500, 1000, 2000, 4000, and 8000 Hz. There is a clear relationship between intelligibility and temporal envelope correlation, but only within the lower frequency bands, with virtually no correlation between temporal envelope information and intelligibility at 4 and 8 kHz (Fig 4 panels E and F). However, we should qualify that this finding does not mean that speech information may not be extracted from envelopes of filtered waveforms at these higher frequencies, but that given the availability of temporal envelope information at low frequencies, subjects rely primarily on low-frequency cues.

The finding that the highest correlation between temporal envelope cues and intelligibility occurs for the 1 kHz band, aligns well with the results of a study by Greenberg et al. [30]. They suggest that bands in the 750–2350 Hz frequency range carry the most useful intelligibility information despite not containing the most spectral energy. It should be noted that speech is unintelligible when strictly limited to this frequency region, but its intelligibility greatly improves when speech in this band is presented simultaneously with one or more other frequency bands. Furthermore, there is neurological evidence that cortical entrainment to speech occurs primarily at bands in this frequency region [31].

### Spectral and temporal smearing

**Fig 5:**
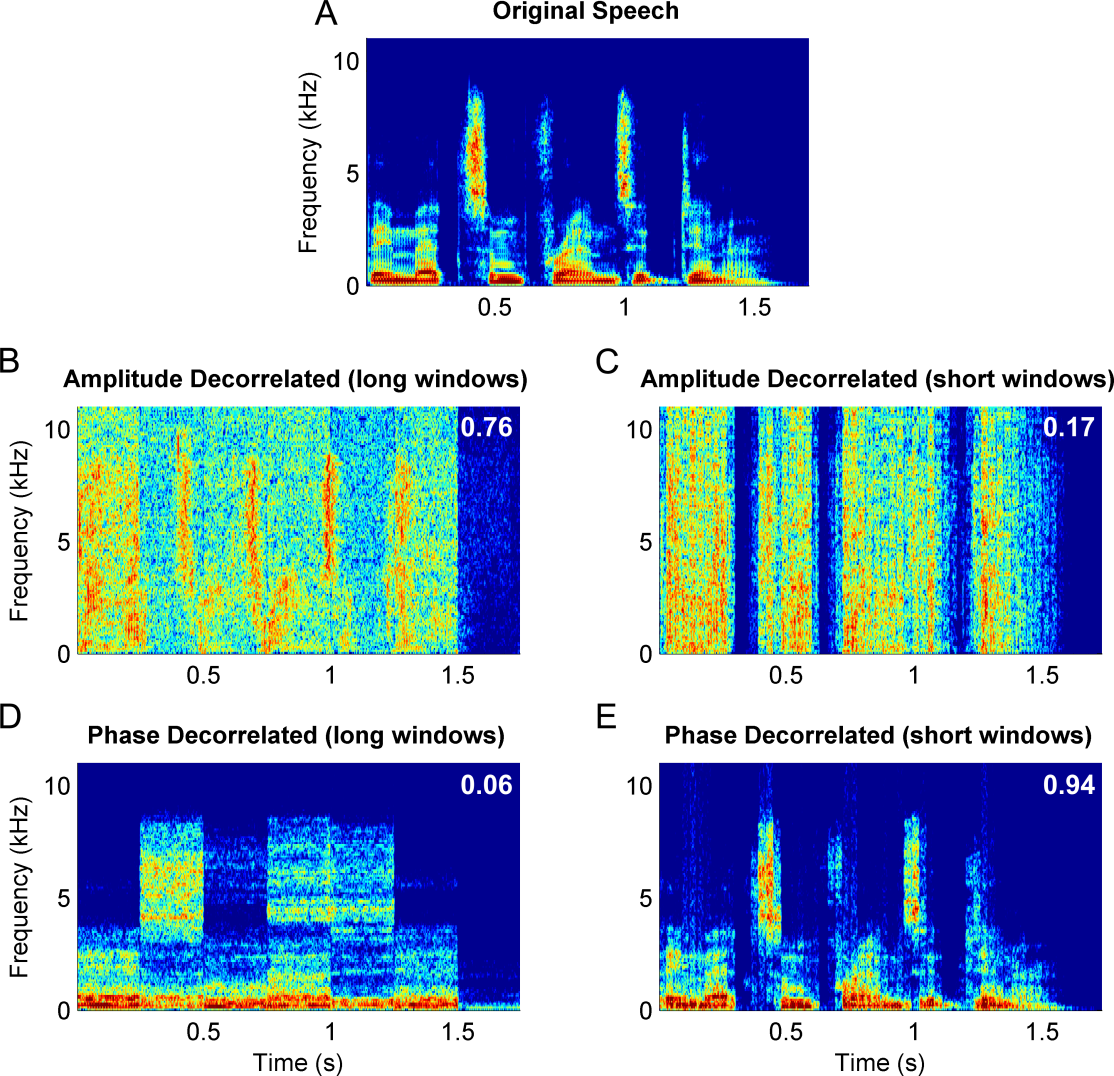
Decorrelated speech spectrograms. Spectrograms for the sentence “They met some friends at dinner.” (A) Original sentence. (B-C) Amplitude spectrum decorrelated with fully correlated phase spectrum (*r*_α_ = 0, *r*_θ_ = 1). (D-E): Phase spectrum decorrelated with unaltered amplitude spectrum (*r*_α_ = 1, *r*_θ_ = 0.4). Left panels show spectrograms for 250 ms (syllable length) windows of analysis, and right panels for 30 ms (phoneme length) windows. The average proportion correct for these parameters are listed on each of the panels.

Spectrograms can be used to visualize the effects of amplitude and phase spectrum decorrelation and help clarify how the decorrelation process degrades temporal and spectral modulations. Fig 5 shows one speech sentence at different levels of decorrelation at two window sizes. We can see that amplitude decorrelation (panels B and C) can be thought of as smearing the energy vertically across frequencies, while phase decorrelation (panels D and E) smears the energy horizontally across time.

With this in mind, it is clear why phase decorrelation significantly affects the intelligibility of sentences segmented into larger (250 ms) windows but less so the shorter ones (30 ms). Phonemes have a roughly 30 ms duration, and therefore when the energy within a 30 ms window is smeared horizontally, the overall change in the phoneme’s energy pattern will be small because it cannot smear as far (it is confined to a brief time window). However, for a 250 ms window length, often encompassing periods of silence as well as several phonemes, smearing along the time axis (horizontally), averages out the energy patterns of several phonemes across time, rendering the speech unintelligible (Fig 5D).

Similarly, when the amplitude spectrum is decorrelated in large time windows, it smears energy across frequencies but allows energy fluctuations across time (such as vowel formants or consonant markers) to remain intact. These intact temporal cues preserve formant information, particularly when processed through cochlear filters, and provide sufficient cues to intelligibility. However, when the analysis window becomes too small (30 ms), formants frequency sweeps will become obscured because the sweep is spread across several windows, allowing sections to be averaged to different levels across time (Fig 5C).

In summary, the current study investigated how amplitude and phase information differentially contribute to speech intelligibility. We found that intelligibility was more adversely affected by phase-spectrum decorrelation than by amplitude-spectrum decorrelation. If the phase information was left intact, decorrelating the amplitude spectrum to intermediate values had no effect on intelligibility. If the amplitude information was left intact, decorrelating the phase spectrum to intermediate values significantly degraded intelligibility. Interestingly, for intermediate amplitude-spectrum correlation values, segment length was generally inconsequential to intelligibility. These findings provide new insights into how spectral degradation in the phase and amplitude domains affect intelligibility, and demonstrate robustness of the processes that code for speech information in environments that acoustically degrade cues to intelligibility.
